# One Health serosurveillance of anti-SARS-CoV-2 antibodies in domestic animals from the metropolitan area of Panama

**DOI:** 10.14202/vetworld.2025.1082-1089

**Published:** 2025-05-08

**Authors:** Sulamith Del C. Pacheco, Alanis J. Jimenez, Giselle A. Rangel, Claudia Del C. Rengifo-Herrera

**Affiliations:** 1 Department of Infectious Diseases and Public Health, Faculty of Veterinary Medicine, University of Panama, Campus Harmodio Arias Madrid, Republic of Panama; 2 Neuroscience Center, Institute of Scientific Research and High Technology Services of Panama (INDICASAT-AIP), Building 208 City of Knowledge, Clayton Panama, Republic of Panama; 3 Sistema Nacional de Investigación, Secretaría Nacional de Ciencia Tecnología e Innovación, Panama City, Panama

**Keywords:** cats, dogs, One Health, Panama, serosurveillance, Severe acute respiratory syndrome coronavirus 2, anthropozoonosis

## Abstract

**Background and Aim:**

The COVID-19 pandemic, caused by Severe Acute Respiratory Syndrome Coronavirus 2 (SARS-CoV-2), has raised concern regarding anthropozoonotic transmission to domestic animals, posing potential public and veterinary health risks. Latin America remains underrepresented in seroepidemiological assessments of such zoonotic spillover. This study aimed to detect anti-SARS-CoV-2 antibodies in domestic dogs and cats in Panama using a One Health surveillance framework.

**Materials and Methods:**

A cross-sectional serological survey was conducted between October 2022 and December 2023 across the metropolitan area of Panama City. Serum samples from 341 animals (198 dogs and 143 cats) were analyzed using a commercial double-antigen enzyme-linked immunosorbent assay to detect antibodies targeting the SARS-CoV-2 nucleocapsid protein. A historical panel of 100 pre-pandemic canine and feline samples was also tested. Demographic, clinical, and exposure data were collected through owner questionnaires, and statistical associations with seropositivity were assessed using univariate tests and binary logistic regression.

**Results:**

Seropositivity was detected in 12/341 animals (3.5%; 95% confidence interval: 1.96–6.11%), comprising 9 dogs (4.5%) and 3 cats (2.1%). In addition, 2/100 pre-pandemic canine samples (2.0%) tested positive. Most seropositive animals (75%) were reported to have lived in households with confirmed COVID-19 cases, although this variable was not statistically associated with seropositivity. Regression analysis identified ideal body condition as a significant predictor (p = 0.016), while sampling location and demographic variables were not significant.

**Conclusion:**

This study presents the first serological evidence of SARS-CoV-2 exposure in domestic pets in Panama. While low in prevalence, the findings underscore the relevance of community-based animal surveillance and reveal possible serological cross-reactivity with endemic canine coronaviruses. The data support the continued integration of domestic animal monitoring into One Health strategies to preempt zoonotic risks and improve pandemic preparedness.

## INTRODUCTION

Severe Acute Respiratory Syndrome Coronavirus 2 (SARS-CoV-2), the etiological agent of COVID-19, belongs to the family *Coronaviridae*, subfamily *Coronavirinae*, and the genus *Betacoronavirus* [1]. This virus encodes several structural proteins, including the membrane protein, nucleocapsid (N) protein, envelope protein, and spike (S) glycoprotein [2]. The S protein facilitates viral entry into host cells and is, therefore, a primary target for vaccine development and serological assays [3]. In contrast, the N protein is highly conserved and abundantly produced during infection [4]. Both proteins play essential roles in viral detection strategies.

SARS-CoV-2 is primarily transmitted through respiratory droplets and aerosols, with an average incu-bation period of 4–5 days. Although some individuals remain asymptomatic, the majority experience mild-to-moderate respiratory symptoms such as cough, fever, headache, myalgia, and diarrhea [2, 5]. Importantly, SARS-CoV-2 can also be transmitted from humans to animals through direct contact – particularly airborne droplets – or potentially through contaminated sur-faces [6]. The virus disseminated rapidly, turning COVID-19 into a global pandemic that overwhelmed public health systems and prompted urgent inquiries regarding its transmission dynamics, immunity, and the potential involvement of various animal species in its epidemiological cycle [7].

The first documented case of natural SARS-CoV-2 infection in an animal occurred in a dog with underlying health issues in Hong Kong in late February 2020; the animal belonged to a COVID-19-positive individual [8]. As of the most recent reports, SARS-CoV-2 infections in animals have been recorded in 29 species across 36 countries spanning the Americas, Africa, Asia, and Europe, according to the World Organization for Animal Health [9]. The demonstrated susceptibility of animals to SARS-CoV-2 raises concerns that certain species could serve as reservoirs for the virus [10–12]. Nevertheless, current data suggest that the likelihood of animal-to-human transmission remains low [13].

Despite increasing global attention to SARS-CoV-2 infections in animals, Latin America remains under-represented in seroprevalence research. The present study seeks to address this gap by generating essential epidemiological insights into SARS-CoV-2 exposure in domestic animals in Panama, thereby supporting regional One Health strategies.

Efforts to enhance pandemic prevention and preparedness are increasingly centered on the One Health framework, which emphasizes integrated interventions – including surveillance and risk assessment – across wildlife, livestock, and human populations [14–18]. Numerous countries have imple-mented epidemiological surveillance in domestic and wild animals to better understand SARS-CoV-2 trans-mission in susceptible species [18]. While interest in animal infections has grown globally, seroprevalence studies in Latin America remain scarce. This research contributes to closing that gap by providing foundational epidemiological data on domestic animals in Panama, reinforcing regional One Health efforts [19–21].

Despite the growing global recognition of the potential role of domestic animals in the transmission dynamics of SARS-CoV-2, there remains a substantial paucity of data from Latin America. Most seroprevalence studies have been concentrated in North America, Europe, and parts of Asia, with limited representation from tropical and low-resource settings such as Panama. Moreover, the majority of existing studies focus on experimentally infected animals or those from high-exposure households, thereby limiting generalizability to broader community settings. In addition, questions regarding species-specific susceptibility, the duration of the antibody response, and the impact of clinical or demographic variables remain largely unexplored in Latin American contexts.

This study aimed to assess the seroprevalence of antibodies against the SARS-CoV-2 N protein in domestic dogs and cats residing in the metropolitan area of Panama City. Using a One Health serosurveillance framework, the study further sought to examine potential associations between seropositivity and demographic, clinical, and exposure-related variables, thereby contributing novel data to the regional understanding of zoonotic spillover and supporting integrated surveillance strategies for pandemic preparedness.

## MATERIALS AND METHODS

### Ethical approval

Ethical approval for this study was granted by the Institutional Animal Care and Use Committee (CICUA) of the Instituto de Investigaciones Científicas y Servicios de Alta Tecnología de Panamá (INDICASAT-AIP), under protocol number CICUA-22-001. The study strictly adhered to ethical standards and implemented a robust, community-based serosurveillance model, which has seldom been applied in Latin American contexts for investigating SARS-CoV-2 in animals. Pet owners provided written informed consent before participation.

### Study period and location

The study was conducted from October 2022 to December 2023 on domestic animals (canines and felines) that attended veterinary clinics and recruitment sessions in the Panama City metropolitan area. The processing and analysis of blood samples were cond-ucted at INDICASAT-AIP laboratories.

### Study design

A cross-sectional design with a single time point measurement was employed. Animals were recruited using stratified probability sampling based on exposure status.

### Study population

The study comprised a serological evaluation of 341 serum samples obtained from domestic dogs and cats residing in the metropolitan area of Panama City. Animals were included regardless of prior exposure to SARS-CoV-2-infected humans. An exposed animal was defined as one that had close contact with a confirmed SARS-CoV-2-positive individual or had resided in a household under quarantine due to infe-ction. Conversely, unexposed animals were those for which owners reported no known contact with SARS-CoV-2-positive individuals or residence in an infe-cted household.

### Study animals recruitment

Dogs and cats were recruited from various veteri-nary clinics and through community outreach efforts conducted at multiple study sites. Recruitment began with the signing of an informed consent form by the pet owner, followed by the collection of demographic and health data using a structured questionnaire, which also included information on SARS-CoV-2 exposure. A standard physical examination of each animal was performed before sample collection.

Eligible animals included domestic dogs and cats in apparent good health, aged ≥3 months, and weighing ≥4 kg. Animals exhibiting aggressive behavior, those with visible clinical alterations, or those undergoing intensive or post-surgical care were excluded from the study.

### Type and handling of samples

All blood samples were collected by licensed veterinary practitioners or trained assistants following established good clinical practices [22]. Approximately 1–2 mL of peripheral blood was drawn through veni-puncture of the cephalic, saphenous, or jugular vein, depending on species and animal size. Samples were centrifuged at 208 × *g* for 10 min, and the resulting serum was aliquoted and stored at –80°C until further analysis.

### Anti-SARS-CoV-2 antibodies detection

Detection of antibodies against the SARS-CoV-2 nucleocapsid (N) protein was performed using a com-mercial double-antigen, multi-species enzyme-linked immunosorbent assay (ELISA) (ID Screen, IDVET, France). According to the manufacturer, this assay demonstrates 100% sensitivity and specificity [23, 24].

### Statistical analysis

Collected data were analyzed using the SPSS version 25.0 (IBM Corp., Armonk, NY, USA) and GraphPad Prism version 8.0 (GraphPad Software, La Jolla, CA, USA). Descriptive statistics were used to summarize categorical variables as frequencies and percentages. Pearson’s Chi-square test was applied to evaluate associations between categorical variables, while analysis of variance (ANOVA) was used for continuous variables. Binary logistic regression was employed to assess the strength of associations and identify potential predictors of seropositivity.

## RESULTS

### Distribution and descriptive characteristics of the recruited animals

A total of 341 animals were enrolled in the study, comprising 198 dogs (58.1%) and 143 cats (41.9%). Among these, 49.6% were female and 48.7% were male. In addition, 60.7% of the animals were classified as mixed breed (MB), including 79/198 dogs (39.9%) and 119/143 cats (83.2%). The mean age of the animals was 5.5 years (±3.7). According to owner responses, 231/341 (72.6%) households reported confirmed COVID-19 cases, and 218/341 (68.6%) indicated that more than 3 months had elapsed since exposure to COVID-19-positive individuals. Regarding lifestyle, 161/341 owners (50.9%) reported that their pets were primarily kept indoors. Moreover, most animals were reported to be free of chronic conditions (255/341, 81.0%) and to have exhibited no respiratory symptoms within the 3 months preceding sampling (292/341, 93.0%) (Table 1).

**Table 1:** Sociodemographic characteristics, COVID-19 exposure, and clinical characteristics of the participants.

Variables	Total n = 341	Species	

		Canine n = 198 (58.1%)	Feline n = 143 (41.9%)
Sex			
Female	169 (50.4)	104 (52.5)	65 (45.4)
Male	166 (49.6)	92 (46.5)	74 (51.7)
Age (years)			
Mean (±SD)	5.5 (±3.7)	5.11 (±3.4)	6.11 (±4.1)
Breed			
Mixed breed	198 (60.7)	79 (39.9)	119 (83.2)
Others	128 (39.3)	114 (57.6)	14 (9.8)
Area of residence			
Panama city	266 (78.0)	164 (82.8)	102 (71.3)
San Miguelito	50 (14.6)	30 (15.1)	20 (13.9)
Cohabit with other pets			
Yes	242 (76.1)	127 (64.1)	115 (80.4)
No	98 (30.8)	70 (35.3)	28 (19.6)
Cohabiting species			
Canine	100 (31.6)	94 (47.5)	6 (4.2)
Feline	87 (27.5)	5 (2.5)	82 (57.3)
Boths	54 (17.1)	27 (13.6)	27 (18.9)
Case of COVID-19 in the household			
Yes	231 (72.6)	145 (73.2)	86 (60.1)
No	87 (27.4)	47(23.7)	40 (28.0)
≥3 months since COVID-19 case at home			
Yes	218 (68.6)	142 (71.7)	76 (53.1)
No or do not know	14 (4.4)	3 (1.5)	11 (7.7)
Pet contact with COVID-19 case			
Yes	173 (54.2)	107 (54.0)	66 (46.1)
No	60 (18.8)	38 (19.2)	22 (15.4)
Lifestyle			
Do not leave home	161 (50.9)	63 (31.8)	98 (68.5)
Exits, but no contact	76 (24.1)	71 (35.9)	5 (3.5)
Exists and has contact	50 (15.8)	44 (22.2)	6 (4.2)
Exits without supervision	29 (9.2)	12 (6.1)	17 (11.9)
Chronic disease			
Yes	60 (19.0)	40 (20.2)	20 (14.0)
No	255 (81.0)	151 (76.3)	104 (72.7)
Symptomatology in the past 3 months			
Yes	22 (7.0)	14 7.1)	8 (5.6)
No	292 (93.0)	176 (88.9)	116 (81.1)

### Serological status for SARS-CoV-2

**Figure 1:**
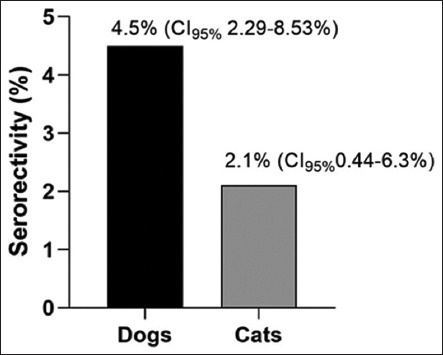
Reactive samples by species. The bars represent the number of subjects evaluated per species that tested positive (reactive) in the assay for detecting anti-severe acute respiratory syndrome coronavirus 2 antibodies in animals.

Antibodies specific to SARS-CoV-2 nucleocapsid (N) protein were detected in 12/341 animals, corres-ponding to a seroprevalence of 3.5% (95% confidence interval [CI]: 1.96%–6.11%) as determined by ELISA. Of these seropositive animals, 9 were dogs (4.5%, 95% CI: 2.29%–8.53%) and 3 were cats (2.1%, 95% CI: 0.44–6.3%) (Figure 1). In addition, among the prepa-ndemic control samples, 2/100 (2.0%, 95% CI: 0.11%–7.44%) canine specimens tested positive.

### Descriptive analysis of seropositive subjects

The sociodemographic, exposure, and clinical characteristics of the seropositive animals are detailed in Table 2. Among the nine seropositive dogs, 5 (55.56%) were female and 4 (44.44%) were male. All three seropositive cats were male. Of the positive canines, 3 (33.3%) were mixed breed, while the remainder included 2 Golden Retrievers (22.2%) and 1 each of Pug, Beagle, Pitbull, and Schnauzer (11.1% each). All seropositive cats were domestic shorthair. The mean age of seropositive animals was 5.33 years (±2.87). Two of the 12 animals (16.67%) had documented chronic diseases, including atopic dermatitis and hepatic lipidosis.

**Table 2:** Sociodemographic, COVID-19 exposure, and clinical characteristics of SARS-CoV-2 seropositive subjects.

Variables	Total, n (%) n = 12
Sex	
Female	5 (41.67)
Male	7 (58.33)
Age (years)	
Mean (±SD)	5.33 (±2.87)
Breed	
Mixed breed	6 (50)
Other	6 (50)
Area of residence	
Panama	12 (100)
San Miguelito	0 (0)
Cohabits with other pets	
Yes	8 (66.67)
No	4 (33.33)
Species cohabiting	
Canine	5 (62.5)
Feline	3 (37.5)
COVID-19 case in the household	
Yes	9 (75.0)
No	3 (25.0)
≥3 months since COVID-19 case in the household	
Yes	8 (88.89)
No	1 (11.11)
Pet’s contact with COVID-19	
Yes	8 (88.89)
No	1 (11.11)
Lifestyle	
Do not leave home	4 (33.33)
Exits, but no contact	5 (41.67)
Exists and has contact	2 (16.67)
Exits without supervision	1 (8.33)
Chronic disease	
Yes	2 (16.67)
No	10 (83.33)
Symptoms in the past 3 months	
Yes	0 (0.0)
No	12 (100.0)

All seropositive animals were located within Panama City. Their distribution included 4 (33.3%) in San Francisco County, 2 (16.7%) each in Parque Lefevre and Bethania Counties, and 1 (8.3%) each in Pueblo Nuevo, Ancon, Bella Vista, and Santa Ana Counties.

With respect to body condition, 7/12 animals (58.3%) were in ideal condition, while 1 (8.3%) was underweight, and another 1 (8.3%) was classified as overweight or obese. Overall health status was con-sidered adequate across all seropositive animals.

### Association between SARS-CoV-2 seropositivity and potential risk factors

To identify variables associated with seropositivity, ANOVA was performed for continuous variables and Pearson’s Chi-square test was applied to categorical variables between seropositive and seronegative SARS-CoV-2 subjects. The results, presented in Table 3, revealed statistically difference with body condition (χ² = 14.695, p = 0.005) and sampling location (χ² = 32.433, p = 0.000).

**Table 3:** Analysis of differences with serologic reactivity to SARS-CoV-2 in domestic animals

Variables	F o x2	p-value
Species	1.437	0.231
Age	0.020	0.887
Gender	0.371	0.542
Breed	25.934	0.767
Chronic diseases	0.048	0.826
Area of residence	16.803	0.965
Cohabits with other pets	0.747	0.387
Number of animals cohabiting	0.937	0.334
Species cohabiting	3.235	0.519
Lifestyle	2.382	0.497
COVID-19 case in the household	0.029	0.866
≥3 months since COVID-19 case in the household	0.457	0.796
Pet’s contact with COVID-19 case	1.092	0.579
Symptoms in the past 3 months	0.943	0.331
Body condition	14.695	**0.005**
Body temperature	0.191	0.662
Heart rate	0.036	0.849
Respiratory rate	0.206	0.650
Capillary refill time	0.421	0.517
Sample collection site	32.433	**0.000**

Significant results are highlighted in bold with p *<* 0.05.

Subsequently, binary logistic regression was conducted to assess the predictive value of body cond-ition and sampling site. The model correctly classified 96.7% of the cases and was implemented in two steps. In the first step, age, species, and sex were entered; in the second step, sampling location and body condition were added. Wald statistics from step one indicated no significant effect of age, species, or sex on seropositivity. In step two, sampling location remained non-significant, whereas body condition – specifically ideal weight and overweight – demonstrated statistical significance in predicting seropositivity (p = 0.016 and 0.052, respectively). These findings are presented in Table 4.

**Table 4:** Binary logistic regression analysis of factors associated with serologic reactivity to SARS-CoV-2 in domestic animals (n = 341).

Variables	b	Standard error	β	p
Step 1				
Age	-0.044	0.095	0.957	0.645
Species	-0.362	0.705	0.696	0.608
Gender	0.497	0.661	1.644	0.452
Step 2				
Age	-0.031	0.099	0.969	0.754
Species	-0.473	0.751	0.623	0.529
Gender	0.575	0.685	1.776	0.402
Location of sample collection				
Site 1	21.279	16374.358	1744021761.433	0.999
Site 2	17.466	16374.358	38500296.873	0.999
Site 3	19.027	16374.358	183354315.795	0.999
Site 4	17.375	16374.358	35155021.055	0.999
Site 5	0.071	16948.442	1.074	1.000
Site 6	0.164	24283.812	1.178	1.000
Body condition				
Thin	-3.152	1.765	0.043	0.074
Low weight	-21.217	10300.722	0.000	0.998
Ideal weight	-3.572	1.484	0.028	**0.016**
Overweight	-3.405	1.750	0.033	0.052

Significant results are highlighted in bold with p *<* 0.05.

## DISCUSSION

In this serological study, 3.5% of the tested animals were positive for specific SARS-CoV-2 antibodies using a double-antigen ELISA, representing the first evidence of SARS-CoV-2 seroreactivity in domestic dogs and cats from the metropolitan area of Panama City. This finding aligns with previous reports of natural SARS-CoV-2 infections in dogs and cats from other countries, such as Mexico [25], USA [26], Canada [27], and Switzerland [28]. It marks the first documentation of SARS-CoV-2 antibody presence in domestic animals in Panama, complementing global data and offering new insights into community-based pet exposure.

A large-scale seroprevalence study conducted by Udom *et al*. [29] in Thailand reported that 35/2103 dogs (1.66%) and 4/1,112 cats (0.36%) tested positive for SARS-CoV-2 antibodies. Consistent with our findings, antibody prevalence was higher in dogs (4.5%) than in cats (2.1%). This may reflect closer physical interaction with infected individuals and/or greater environmental exposure in dogs compared to cats.

These findings stand in contrast to the experimental study by Shi *et al*. [30] that suggests lower susceptibility in dogs and higher susceptibility in cats. Our results indicate that although cats may be more susceptible under laboratory conditions, dogs may experience higher exposure and infection rates in real-world community settings, likely due to behavioral and environmental factors. This discrepancy underscores the need for further investigation into host-specific susceptibility factors.

Zhang *et al*. [31] have reported divergent findings, such as 15 out of 102 cats (14.7%) in Wuhan testing positive during the SARS-CoV-2 outbreak, whereas a separate study by Deng *et al*. [32] involving 423 cats from various Chinese cities found no seropositive cases. These variations suggest that environmental and behavioral factors may play a more critical role than species-specific susceptibility in SARS-CoV-2 transmission to pets. Supporting this, research has demonstrated efficient direct transmission of SARS-CoV-2 among cats, with limited transmission through indirect environmental exposure [33].

Close contact with COVID-19-infected individuals has been examined in several studies as a potential risk factor for pet seroconversion. A German study by Michelitsch *et al*. [34] involving only animals from COVID-19-positive households found high sero-prevalence rates in both cats (41.7%) and dogs (55.9%), supporting the hypothesis that household exposure is a primary source of infection for pets [34]. However, our findings diverge from this pattern. Although 72.6% of the recruited animals came from households with a history of COVID-19 and 54.2% had documented contact with infected individuals during convalescence, no statistically significant association between household exposure and seropositivity was observed. These results challenge the prevailing assumption that close human-animal contact alone is a major determinant of SARS-CoV-2 transmission to pets.

Regarding the humoral immune response in animals, Zhang *et al*. [31] have suggested that SARS-CoV-2 antibody levels in domestic species wane significantly within approximately 3 months. Given that 68.6% of our study population had exposure more than 3 months before sampling, it is plausible that antibody titers had declined, leading to an underestimation of seropositivity in our findings.

A significant association was identified between body condition and ELISA reactivity, with animals in the ideal to overweight range exhibiting a higher likelihood of seropositivity. Newberne [35] have shown that overfed dogs are more susceptible to infections such as canine distemper and canine adenovirus type 1. While severe obesity and overfeeding have been linked to immunosuppression, the underlying mechanisms remain poorly understood.

Reports of naturally occurring SARS-CoV-2 infection in pets are limited, and severe clinical manifestations are infrequent. When complications are observed, comorbidities often appear to be contributing factors [8]. According to the World Organization for Animal Health, fewer than half of infected animals exhibit clinical signs such as fever, cough, respiratory distress, lethargy, sneezing, nasal and ocular discharge, vomiting, or diarrhea [36]. In this study, none of the 12 seropositive animals displayed clinical signs in the 3 months preceding sampling, and only 2 (16.7%) had chronic medical conditions. Although these variables did not correlate with seropositivity, the clinical profiles observed are consistent with current literature indicating that most infected pets remain asymptomatic.

This study also evaluated the presence of other pets, either of the same or different species, in seropositive households. None of the positive cases resided in the same household, suggesting that SARS-CoV-2 transmission in domestic environments may be self-limiting.

## CONCLUSION

This study provides the first serological evidence of SARS-CoV-2 exposure in domestic dogs and cats in Panama, with an overall seroprevalence of 3.5% among animals tested using a double-antigen ELISA. Seropositivity was higher in dogs (4.5%) than in cats (2.1%), and all seropositive animals were asymptomatic. A significant association was observed between body condition and antibody reactivity, suggesting potential immunological or metabolic factors influencing susc-eptibility. Contrary to previous assumptions, no signi-ficant correlation was found between household COVID-19 exposure and pet seropositivity, highlighting the need to reevaluate existing paradigms of zoonotic transmission dynamics in domestic settings.

A major strength of this study lies in its One Health approach, employing community-based surveillance and including a broad population of household pets with and without documented human exposure. In addition, the inclusion of prepandemic control samples added a critical baseline for interpreting serological findings. However, the study is limited by its cross-sectional design, which precludes causal inference and temporal evaluation of infection. Furthermore, the lack of molecular confirmation (e.g., reverse tran-scription polymerase chain reaction) and potential cross-reactivity with endemic animal coronaviruses may influence the specificity of serological results.

Future studies should incorporate longitudinal sampling, viral detection, and genomic analyses to confirm active infection and elucidate transmission pathways. Expanding surveillance to include rural and peri-urban populations, as well as integrating wildlife and livestock monitoring, will be essential for a more comprehensive understanding of SARS-CoV-2 ecology. These efforts are vital for informing risk assessment and response strategies under the One Health framework and strengthening preparedness for future zoonotic outbreaks.

## AUTHORS’ CONTRIBUTIONS

GAR and CCR: Conceptualized, designed, and supervised the study. SCP and AJJ: Collected blood samples, applied questionnaires, and performed the ELISA tests. GAR: Analyzed the data and graphic constructions. GAR, SCP, and AJJ: Wrote sections of the manuscript. CCR: Revised the manuscript for intellectual and scientific content. All authors have reviewed the manuscript and agreed to its final version.
